# Full-Genomic Analysis of a Human Norovirus Recombinant GII.12/13 Novel Strain Isolated from South Korea

**DOI:** 10.1371/journal.pone.0085063

**Published:** 2013-12-31

**Authors:** Yu-Jung Won, Jeong-Woong Park, Sang-ha Han, Han-Gil Cho, Lae-Hyung Kang, Sung-Geun Lee, Sang-Ryeol Ryu, Soon-Young Paik

**Affiliations:** 1 Department of Microbiology, College of Medicine, The Catholic University of Korea, Seoul, Republic of Korea; 2 Department of Food and Animal Biotechnology, Department of Agricultural Biotechnology, Center for Agricultural Biomaterials, and Research Institute for Agriculture and Life Sciences, Seoul National University, Seoul, Republic of Korea; 3 Division of Virology, Gyeonggi Provincial Research Institute of Public Health and Environment, Suwon, Republic of Korea; 4 Korea Zoonosis Research Institute, Chonbuk National University, Jeonju, Republic of Korea; University of Malaya, Malaysia

## Abstract

Norovirus (NoV) genogroups I and II are frequently recognized as the main causes of acute gastroenteritis and outbreaks of non-bacterial foodborne diseases. Furthermore, variants and recombinant strains of this virus are continuously emerging worldwide. The aim of this study was to identify NoV strains and to investigate and characterize rare genotypes. Stool samples (*n* = 500) were collected from patients with symptoms of acute gastroenteritis in Korea between December 2004 and November 2007. For analysis of the samples, rapid genotype screening was performed using reverse transcriptase-polymerase chain reaction. Full sequencing, using a newly designed set of 12 primers, revealed GII-12/13 strain. The partial sequence of GII-12/13 strain was compared with published NoV (GII-1 - 14) sequences targeting RdRp and capsid regions using phylogenetic analysis with the SimPlot program, which could evaluate recombination breakpoints. SimPlot analysis was also performed with the strain GII-12/Gifu-96/JPN (AB045603) for the RdRp region and with GII-13/G5175B-83/AUS(DQ379714) for the capsid region. NoV was detected in 19 of the 500 stool samples (3.8%). Genogroup GII-4 was found most frequently (*n* = 9, 1.8%), followed by GII-3 (*n* = 4, 0.8%), GII-6 (*n* = 3, 0.6%), GI-6 (*n* = 2, 0.4%), and GII-12/13 (*n* = 1, 0.2%). Importantly, we identified a novel NoV recombinant strain, C9-439 (KF289337), indicating potential risks, which suggested that, recombination occurred in the region between open reading frames 1 and 2 of the GII-12/13 strain and that breakpoints occurred in the polymerase region.

## Introduction

Norovirus (NoV) is recognized as a major etiologic agent of nonbacterial acute gastroenteritis in all age groups worldwide [1]. The symptoms of acute gastroenteritis due to NoV infection are typically disappeared within 48 h. Indeed, the symptoms of NoV infections are generally mild and self-limiting; adults usually recover after mild diarrhea, but it could be life-threatening in immunocompromised patients [2]. NoV infections are estimated to kill approximately 200,000 children under the age of 5 years each year in developing countries [3]. In developed countries, mortality due to viral gastroenteritis is infrequent, but morbidity and economic consequences are nonetheless significant [4]. To date, many cases of sporadic infection caused by waterborne and foodborne NoVs have been reported, and acute gastroenteritis due to NoV has become a major public health concern globally. The main route of transmission is not only by contact with an infected individual, but also through contact with the infectious vomitus, contaminated food, water, and environment, or aerosolized virus [5]. Furthermore, epidemics of acute gastroenteritis are closely associated with the emergence of antigenic variants forming a specific genetic relationship. 

NoVs have been classified as a genus within the virus family *Caliciviridae*, and they harbor a positive-sense, single-stranded RNA genome of ~7.5 kb [6]. NoVs are divided into five different groups (GI - GV) based on the sequence of the capsid and RdRp regions, and each group is further divided into several genogroups [7]. Only three genogroups (GI, GII, and GIV) sporadically infect humans [8], and at present, GII-4 is known as the most prevalent genotype in humans, responsible for approximately 60% of NoV outbreaks [9]. The NoV genome consists of three open reading frames (ORFs); ORF1 encodes a large nonstructural polypeptide (Ntpase, p22, VPg, 3CLpro, RdRp), ORF2 encodes the major structural capsid protein (shell, P1, and P2 domains), and ORF3 encodes a minor structural protein [10].

NoVs are widely known to have high rates of recombination based on their patterns of viral evolution [8]. The overlapping region of ORF1/ORF2 is generally considered the most common recombination break-point in most NoV recombinants [11]. Many novel NoV recombination strains, including NVGII.1/NVGII.5 in India [12] and GII3/GIIb in Australia [13], were characterized by their novel RdRp clusters. In South Korea, two recombinant strains, GII-4/GII-3 and GII-b/GII-16, emerged between 2007 and 2008 in Jeju Island [14]. Thus, these discoveries have demonstrated that the RNA recombination of NoVs contributes to the genetic diversity and emergence of new NoV variants. Therefore, since new recombinant strains of NoV may pathogenic mechanisms and virulence characteristics that differ from those of known strain, monitoring the prevalence and emergence of such new strains is essential for public health. However, studies related to the mechanisms of NoV infections are lacking due to difficulties in culturing the virus. Recently, the reverse transcription-polymerase chain reaction (RT-PCR) method has been widely used for the detection and investigation of genetic diversity of NoV [15,16]. RT-PCR amplifies specific fragments of the genome, thereby enabling sensitive and specific genotyping. 

The purpose of this study was to detect and characterize NoV in stool samples obtained from Korean patients with acute gastroenteritis between 2004 and 2007. Extracted RNA from fecal samples was amplified using RT-PCR, and full genomic sequences of isolated strains were compared to those of other known typical NoV strains that have been reported globally to investigate genomic relationships. In addition, this study was to identify NoV recombination strain which was newly detected in South Korea using full genomic sequencing. 

## Material and Methods

### Ethics statement

In this study, samples were collected during the medical treatment of patients with acute gastroenteritis. Written informed consent was provided by all patients and this information has been kept on file at the Catholic University of Korea Incheon St. Mary’s Hospital. There was no human rights abuse or ethical issues during the process of this study. All experimental work and sample collection was supervised by the IRB of Songeui Medical Campus, The Catholic University of Korea (approval number: MC13SASI0077).

### Stool sample preparation

Between 2004 and 2007, 1,337 stool samples were collected from children who were under 5 years of age, had diarrhea. A case of diarrhea for the stool collection in this study was defined as increased stool frequency (i.e., at least three loose and watery stools within a 24 hr period) with or without vomiting occurring within the previous 24 h. In this experiment, the 500 samples (37%) were randomly selected and sent to the Waterborne Virus Bank (Seoul, South Korea) then kept at -80°C. The stool samples were finally transported on dry ice to the Department of Microbiology, Catholic University of Korea for etiology study of NoV. For sample processing, approximately 10% suspensions of stool samples were prepared by vortexing 0.1 g of stool sample with 1 mL of phosphate-buffered saline (PBS; pH 7.2). The stool sample suspensions were centrifuged for 30 min at 13,000 x*g*, and the supernatants were harvested. The pre-treatment samples were stored at −80°C until used for analysis.

### Viral RNA extraction

Viral RNA was extracted from 140 μL of supernatant using the QIAamp Viral RNA mini Kit (Qiagen, Westburg, Germany) according to the manufacturer’s instructions. The RNA was either reverse transcribed immediately or stored at −80°C until use.

### Reverse transcription polymerase chain reaction (RT-PCR)

For detection of NoV from stool samples, RT-PCR was performed with the OneStep RT-PCR system (Qiagen, Hilden, Germany), using primers based on the partial genome sequence of the detected NoV ORF2 region (Table 1). RT-PCR was performed under the following conditions: a reverse transcription step at 50°C for 30 min and PCR involving an initial activation step at 95°C for 15 min, followed by 40 cycles each of 94°C for 1 min, 55°C for 1 min, and 72°C for 1 min, with a final extension step at 72°C for 10 min. The PCR products were then analyzed by electrophoresis on 2% agarose gels stained with ethidium bromide.

**Table 1:** Primer sequences of the RT-PCR assays.

**Genotypes**	**Primer**	**Sequence (5'**→**3'**)	**Region**	**Position**
I	GI-FIM	CTGCCCGAATTYGTAAATGATGAT	ORF1/2	5342-5671**^a^**
	GI-RIM	CCAACCCARCCATTRTACATYTG		
II	GII-FIM	GGGAGGGCGATCGCAATCT	ORF1/2	5049-5389**^b^**
	GII-RIM	CCRCCTGCATRICCRTTRTACAT		

^a^ GenBank accession number M87661.

^b^ GenBank accession number X86557.

### Cloning and sequencing of RT-PCR products

The amplified fragments were extracted from the gel and purified using the HiYield Gel/PCR DNA Extraction Kit (RBC, Taipei, Taiwan). The purified products were then cloned using the qGEM-T Easy vector (Promega, Madison, WI, USA) according to manufacturer’s instructions and incubated overnight at 4°C to obtain the maximum number of transformants. Transformants were selected on Luria-Bertani (LB) agar media (Duchefa; Haarlem, NED) containing 50 μL/mL ampicillin, 0.23 g/mL 1M isopropyl-β-d-thio-galactoside (IPTG), and 40 ng/mL X-gal. Clones were inoculated at 37°C in 10 mL LB media containing 50 μL/mL ampicillin, and incubated overnight at 37°C in a shaking incubator. Plasmid DNA was purified using a HiYield Plasmid Mini Kit (RBC, Taipei, Taiwan), and DNA was sequenced by Cosmogentech, Ltd., South Korea. 

### Phylogenetic analysis

The partial nucleotide sequences of ORF2 genes were aligned, and phylogenetic analysis of the aligned sequences was performed with published NoV strains obtained from the National Center for Biotechnology Information (NCBI) using MegAlign version 4.0. In this study, the sequences of NoV strains GI and GII were previously classified into 25 genotypes as described by Zheng et al. [17]. The reference strain used in this study was from NCBI and is shown in Table 2. Phylogenetic trees were constructed using the neighbor-joining method for DNA sequence alignments and dendrograms. 

**Table 2:** Reference nucleotide sequences for norovirus classification.

**Genotype**	**Strain**	**Accession number**	**Genotype**	**Strain**	**Accession number**
GI_1	NV-USA93	M87661	GII_6	SU3-JPN	AB039776
GI_2	SOV-GBR93	L07418	GII_7	GN273-USA93	AF414409
GI_3	VA98115-USA98	AY038598	GII_8	SaU25-JPN	AB039780
GI_4	B493-GER93	X76716	GII_9	VA97207-USA97	AY038599
GI_5	SzUG1-JPN	AF414406	GII_10	Erfurt-DEU00	AF427118
GI_6	BS5-GER	AF093797	GII_11	SW918-JPN97	AB074893
GI_7	Wnchest-GBR94	AJ277609	GII_12	Gifu-JPN96	AB045603
GI_8	Boxer-USA01	AF538679	GII_13	G5175B-AUS83	DQ379714
GII_1	Hawaii-USA94	U07611	GII_14	8533Maizuru-JPN08	GU017903
GII_2	SMV2-USA	AY134748	GII_15	Me7076-USA99	AF542090
GII_3	SU201-JPN	AB039782	GII_16	Tiffin-USA99	AY502010
GII_4	Lordsdale-UK	X86557	GII_17	CSE1-USA02	AY502009
GII_5	MOH-HUN99	AF397156			

### Full sequencing

To facilitate the sequencing of the full genome of the novel NoV strain, RT-PCR was performed using a One-Step RT-PCR Kit (Qiagen, Hilden, Germany) with 12 pairs of newly designed primer sets (Table 3). The new primers were designed based on the published GII.12 and GII.13 genotypes of NoVs worldwide, including eight fragments for ORF1, three fragments for ORF2, and one fragment for ORF3. Each PCR product was confirmed by amplification through electrophoresis as described above. 

**Table 3:** Newly designed primers used in this study.

**Primer Name**	**Sequence (5'**→**3'**)	**Position (nt)**	**Size (bp)**	**Target region**	**Reference**
ORF1-1F	GTG AAT GAA GAT GGC GTC TA	1-20			
ORF1-1R	AGT CTT GGT AGG GCC TAA AG	697-716	716		
ORF1-2F	GTG TAG GAG AAT GAT CCA G	664-682			
ORF1-2R	GAG AGC TCC TCC TTC GC	1436-1452	789		
ORF1-3F	CCA AGT CTG CTT CAC CTG AC	1353-1372			
ORF1-3R	GGG TGT TTC CGT TCT TGT C	1976-1994	642		
ORF1-4F	ACT GAC GCT GGC TCC GCA	1948-1965			
ORF1-4R	ATC CTT GCT AGC GGT CTC CT	2547-2566	619	ORF1	In this study
ORF1-5F	GTC AAG CGC ATG AAC ATA CAA G	2492-2513			
ORF1-5R	CTC CAG TTG GCC TCT TGA TGA	3285-3305	814		
ORF1-6F	GGC ATG ATC TTG GAA GAA GG	3236-3255			
ORF1-6R	ATG CTT GCG CGA ATG ACC	3882-3899	664		
ORF1-7F	GGC TGC CAA GAA AAC CAT C	3823-3841			
ORF1-7R	ACC TCA GAA AGT GCA CAG AG	4523-4542	720		
ORF1-8F	CCA ATG GAA TTC CAT CGC CC	4489-4508			
ORF1-8R	CGA CGC CAT CTT CAT TCA CA	5080-5099	611		
ORF2-1F	CGC AAT CTG GCT CCC AGT T	5060-5078			
ORF2-1R	GTG CAT CTG CCA TTC TGA CAC	5864-5884	825	ORF2	In this study
ORF2-2F	GAG CAA TTG TAT ACG GCT CC	5823-5842			
ORF2-2R	CTG GAT TCT TCT ACG CCC AT	6691-6710	888		
ORF3-2F	GAA CTG GYA ATG GGC GTA	6682-6699		ORF3	In this study
ORF3-2R	TGC AAG NNN NTN NNN TCA C	7473-7492	811		

### Detection of recombination

The full sequence of the NoV strain were divided into each part (ORF1, 246 bp; ORF2, 483 bp; ORF3, 212 bp), and phylogenetic analysis was performed with the reference strain (Table 2) using MegAlign version 4.0 [18]. 

### SimPlot analysis

The full sequences of the recombination strains were analyzed using the SimPlot ver.4 program to investigate the break-point of the isolated NoV strains. The SimPlot analysis was conducted with the partial 2,036 bp sequences of the ORF1 and ORF2 regions by using a window size of 200 bp and a step size of 20 bp. 

## Results

### NoV detection

In total, 20 samples were identified as NoV positive. The detection rates of NoV GI and GII were 0.4% (2/500) and 3.4% (17/500), respectively. Genome sequences of NoV positive samples were obtained (GI, 330 bp; and GII, 341 bp), and BLAST analysis was performed at the NCBI website. The BLAST search revealed that the GI-6 genotype was present in all isolated GI positive samples (*n* = 2). Among the GII-positive samples (*n* = 17), the GII-3 (*n* = 4), GII-4 (*n* = 9), GII-6 (*n* = 3), and GII-13 genotypes (*n* = 1) were identified. The sequences obtained in this study were registered in NCBI (accession numbers KF289316, KF289317, KF289320 to KF289335, and KF289337).

### Phylogenetic analysis

**Figure 1:**
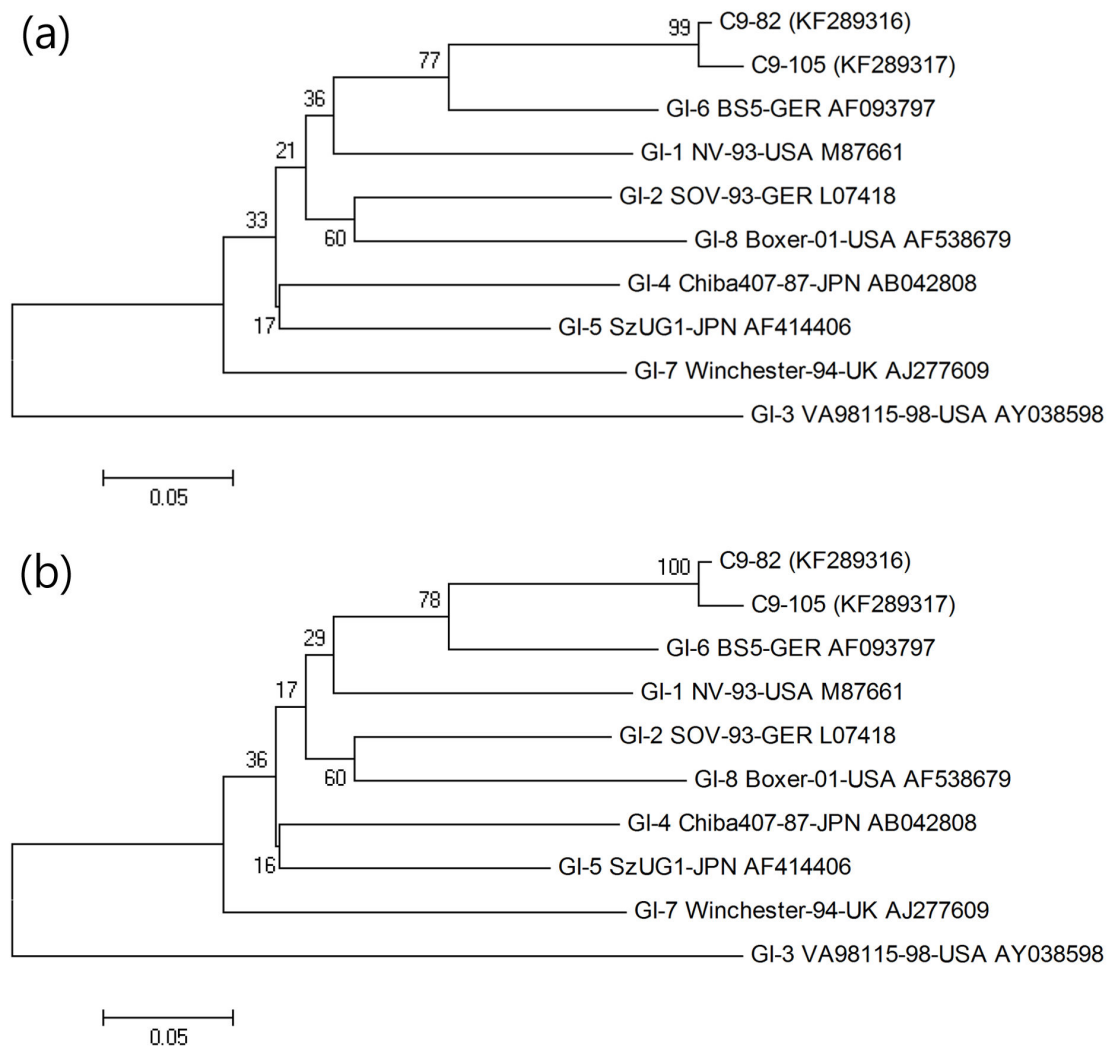
Phylogenetic analysis of the nucleotide (a) and amino acid (b) sequences of the ORF1/ORF2 region of the GI type strain detected through genotyping with the reference strain isolated worldwide.

Phylogenetic analysis was performed to evaluate the genetic relationships among NoV-positive samples that had a 234 bp sequence and other published reference strains (Table 2). In the case of GI, the C9-82 (KF289316) and C9-105 strains (KF289317) showed maximum identity with the GI-6 BS5-GER strain (AF093797), with 83.3% and 83.3% similarity for nucleotide sequences and 96.1% and 94.8% similarity for amino acid sequences, respectively. On the other hand, the C9-82 (KF289316) and C9-105 strains (KF289317) showed lower similarity values to the GI-3 VA98 115-USA strain (AY038598) of 64.5% and 64.5% for nucleotide sequences and 63.6% and 64.9% for amino acid sequences, respectively (Figure 1a and b).

**Figure 2:**
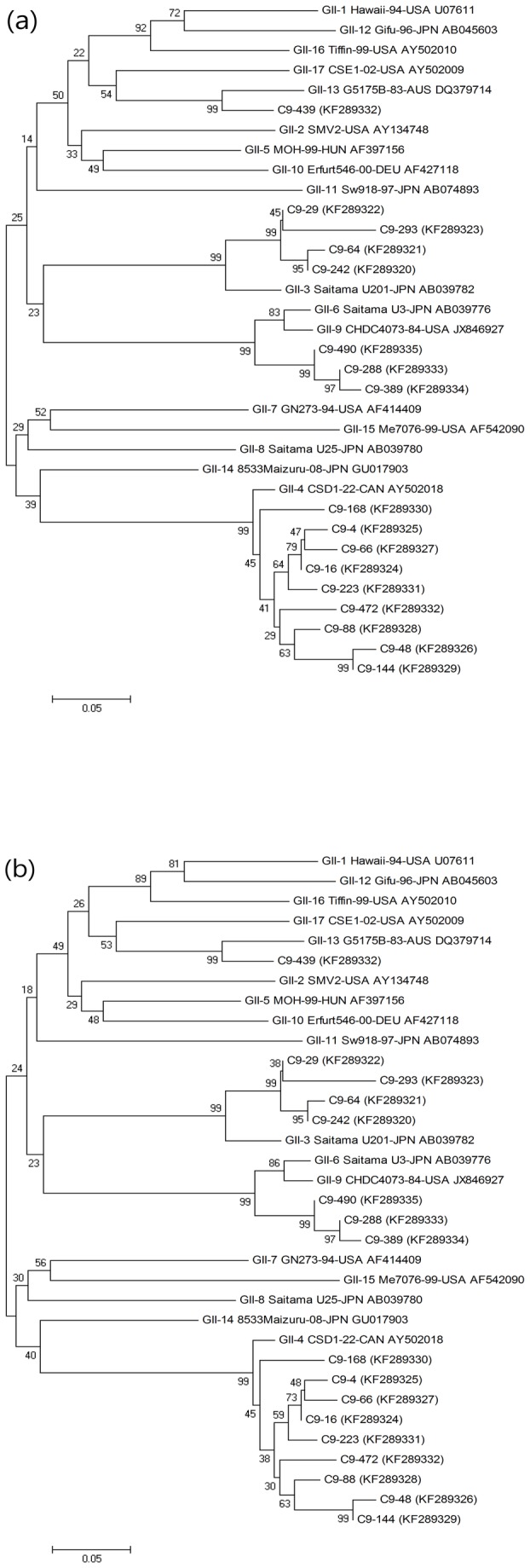
Phylogenetic analysis of the nucleotide (a) and amino acid (b) sequences of the ORF1/ORF2 region of the GII type strain detected through genotyping with the reference strain isolated worldwide.

In the case of GII, the C9-29 (KF289322), C9- 64 (KF289320), C9-242 (KF289320), and C9-293 strains (KF289323) showed maximum identity with the GII-3 Saitama U201-JPN strain (AB1039782) with 92.7%, 90.6%, 91.5%, and 88.9% similarities for nucleotide sequences and 96.1%, 92.2%, 93.5%, and 85.7% similarities for amino acid sequences, respectively. The C9-29 (KF289322), C9-64 (KF289320), C9-242 (KF289320), and C9-293 strains (KF289323) showed relatively lower similarity values to the GII-15 Me7076-99-USA strain (AF542090) with 69.7%, 68.4%, 69.2%, and 66.2% similarity for nucleotide sequences and 72.7%, 70.1%, 70.1%, and 66.2% similarity for amino acid sequences, respectively (Figure 2a and b). 

GII-4 strains (KF289324 to KF289332) were analyzed using the BLAST tool of NCBI. Sequence analysis of GII-4 strains (KF289324 to KF289332) of NoV revealed 93.6 - 96.2% identity for nucleotide sequences and 88.3 - 98.7% identity for amino acid sequences with the GII-4 CDS1-22-CAN strain (AY502018). Compared to the GII-12 Gifu-96-JPN strain (AB045603), the nucleotide sequence identity of GII-4 strains (KF289324 to KF289332) was 63.2 - 68.8%, and the amino acid sequence identity was 66.2 - 75.3% (Figure 2a and b), respectively.

The C9-288 (KF289333), C9-389 (KF289334), and C9-490 (KF289335) strains showed maximum identity with the GII-6 Saitama U3-JPN strain (AB039776), with nucleotide sequence similarities of 91.9%, 90.6%, and 92.7%, respectively, and amino acid sequence similarities of 90.9%, 88.3%, and 94.8%, respectively. In contrast, the C9-288 (KF289333), C9-389 (KF289334), and C9-490 (KF289335) strains showed lower similarity values with the GII-15 Me7076-99-USA strain (AF542090) for nucleotide sequences (66.7%, 66.7%, and 67.5%, respectively) and amino acid sequences (71.4%, 70.1%, and 75.3%, respectively; Figure 2a and b).

The C9-439 strain (KF289337) showed 70.9 - 91.9% nucleotide sequence identity and 77.9 - 98.7% amino acid sequence identity to the reference strain. Among all reference strains, the C9-439 strain was the most similar to the GII-13 G5175B-83-AUS strain (DQ379714), with 91.9% nucleotide sequence identity and 98.7% amino acid sequence identity (Figure 2a and b).

### ORFs analysis

The whole genome of the C9-439 strain (KF289337) was composed of 3 ORF regions: ORF1, 5100 bp; ORF2, 1628 bp; and ORF3, 735 bp. A phylogenetic tree was constructed by sequence analysis of the partial ORF1 (246 bp), ORF2 (483 bp), and ORF3 (212 bp) sequences. The partial nucleotide sequences of the novel NoV strain isolated in this study were analyzed in comparison to the reference strain with MegAlign version 4.0 (Table 2).

#### ORF1 (*nonstructural protein*)

**Figure 3:**
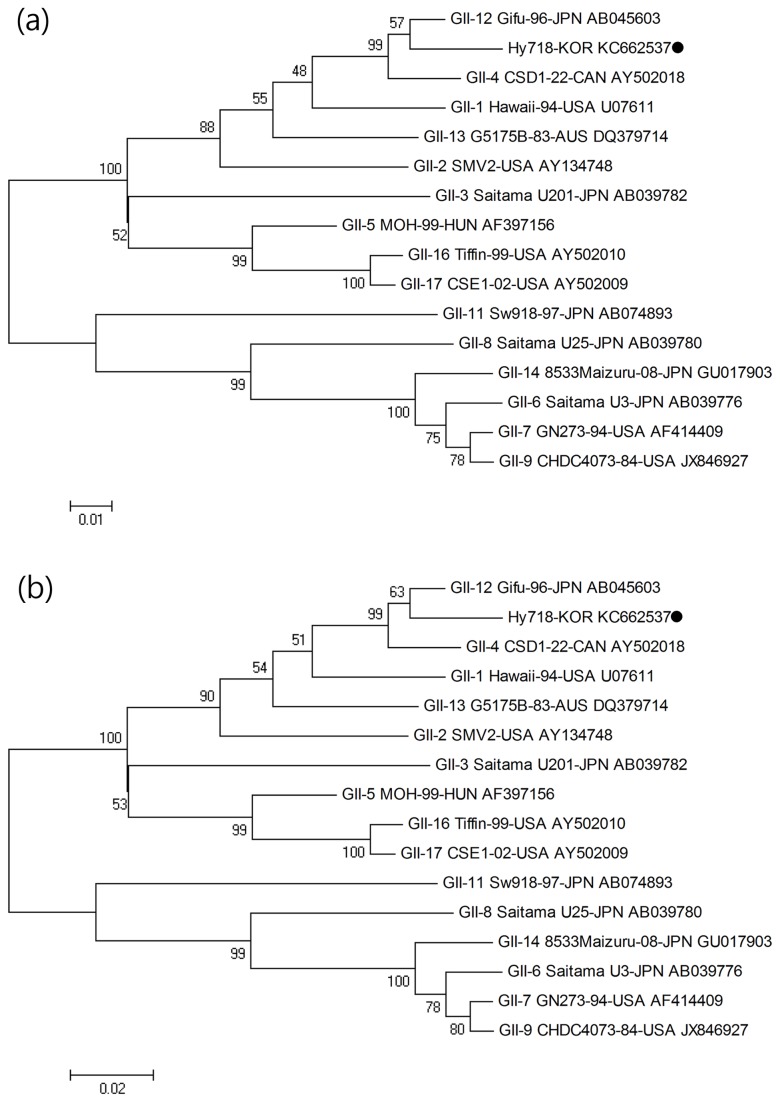
Phylogenetic analysis of the nucleotide (a) and amino acid (b) sequences of the partial ORF1 region (246 bp) of the Hy718-KOR strain (C9-439 sample) and reference strain isolated worldwide. The novel norovirus strain isolated in this study is indicated with ●.

Phylogenetic analysis was performed with the target 246 bp sequence of the ORF1 region of the C9-439 strain and reference strains frequently isolated worldwide (Table 2). With respect to the ORF1 region, the nucleotide and amino acid sequence identities ranged from 70.3% to 95.5% and from 74.1% to 98.8%, respectively. Specifically, the C9-439 strain showed the highest similarity with the GII-12 Gifu-96-JPN strain with 95.5% and 98.8% nucleotide and amino acid sequence identity, respectively (Figure 3a and b). 

#### ORF2 (the *VP1* gene)

**Figure 4:**
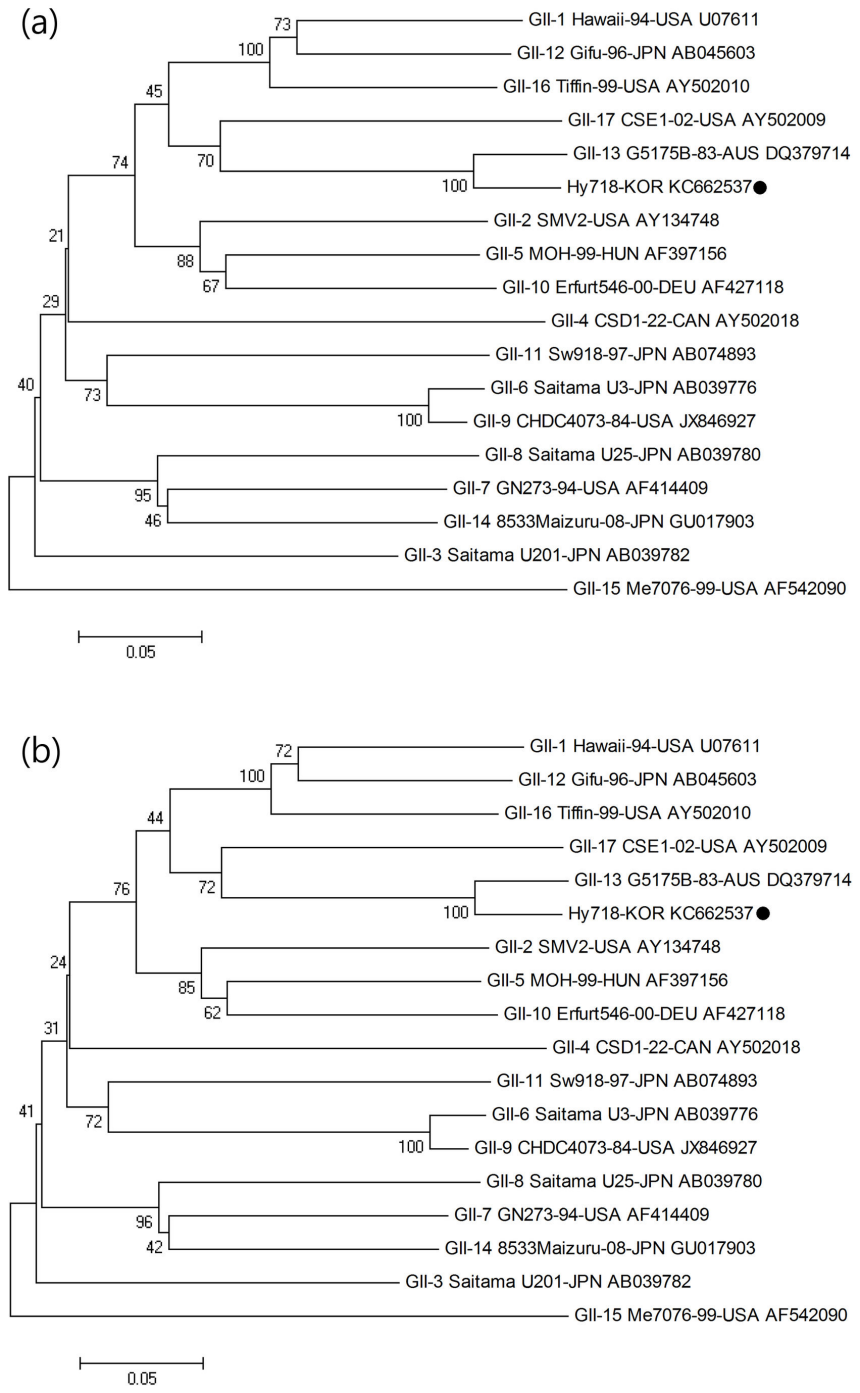
Phylogenetic analysis of the nucleotide (a) and amino acid (b) sequences of the partial ORF2 region (483 bp) of the Hy718-KOR strain (C9-439 sample) and reference strain isolated worldwide. The novel norovirus strain isolated in this study is indicated with ●.

Phylogenetic analysis of the ORF2 region was performed with the target 483 bp sequence and reference strains (Table 2). Nucleotide sequence and amino acid sequence identity with reference strains ranged from 67.3% to 93.0% and from 72.7% to 99.4%, respectively. Specifically, the C9-439 strain showed the highest similarity with the GII-13 G5175B-83-AUS strain (DQ379714), exhibiting 93.0% and 99.4% nucleotide and amino acid sequence identity, respectively (Figure 4a and b).

#### ORF3 (the *VP2* gene)

**Figure 5:**
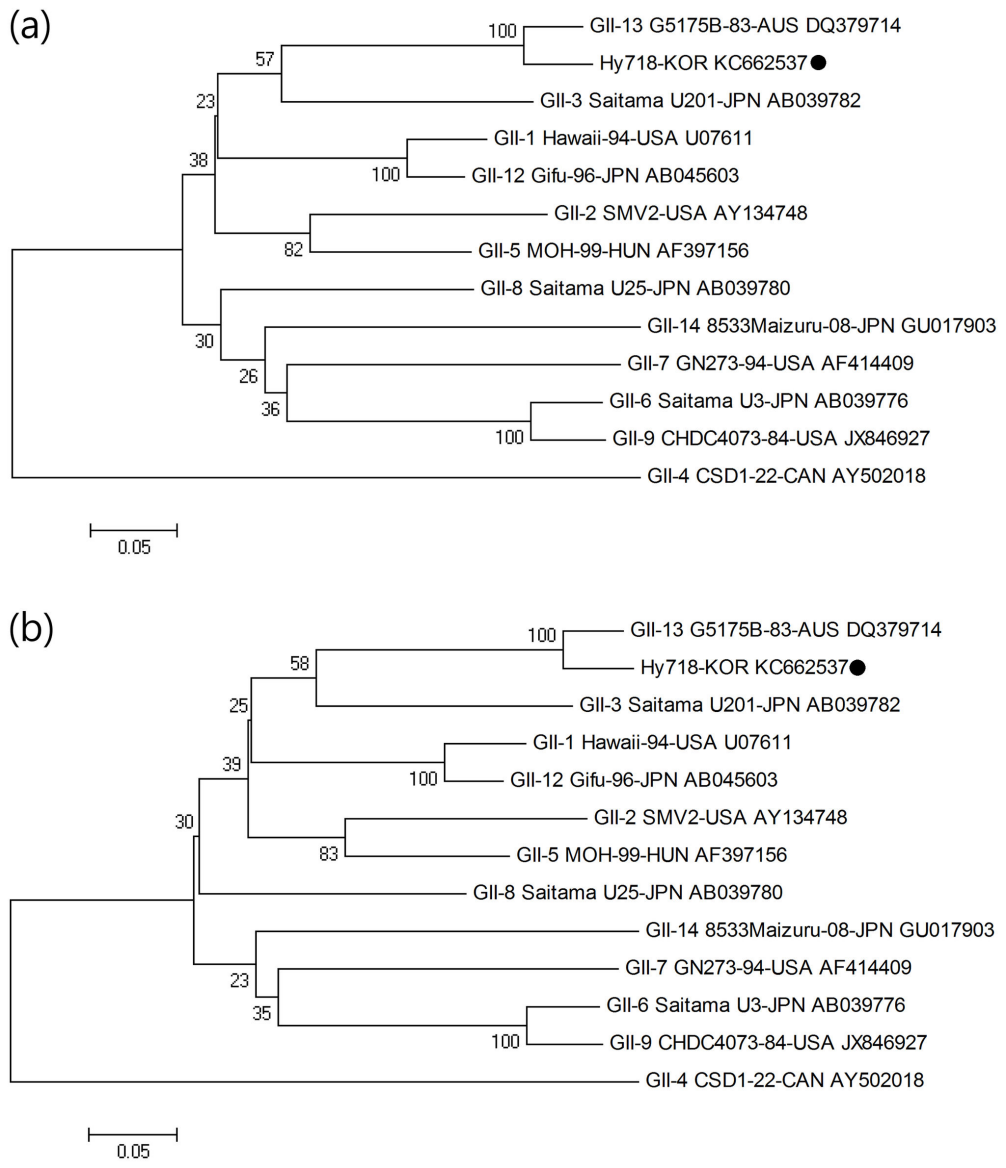
Phylogenetic analysis of the nucleotide (a) and amino acid (b) sequences of the partial ORF3 region (212 bp) of the Hy718-KOR strain (C9-439 sample) and reference strain isolated worldwide. The novel norovirus strain isolated in this study is indicated with ●.

Phylogenetic analysis of ORF3 (the *VP2* gene) is described in Table 2. This analysis targeted 212 bp of the ORF3 region of the C9-439 strain and reference strains isolated worldwide. With respect to the ORF3 region, the nucleotide sequence identity with reference strains of other genotypes ranged from 54.1% to 92.9%. The amino acid sequence identity ranged from 51.4% to 100%. The GII-13 G5175B-83-AUS strain (DQ379714) showed the highest similarity with the C9-439 strain, exhibiting 92.9% and 100% nucleotide and amino acid sequence identity, respectively (Figure 5a and b).

### SimPlot analysis

**Figure 6:**
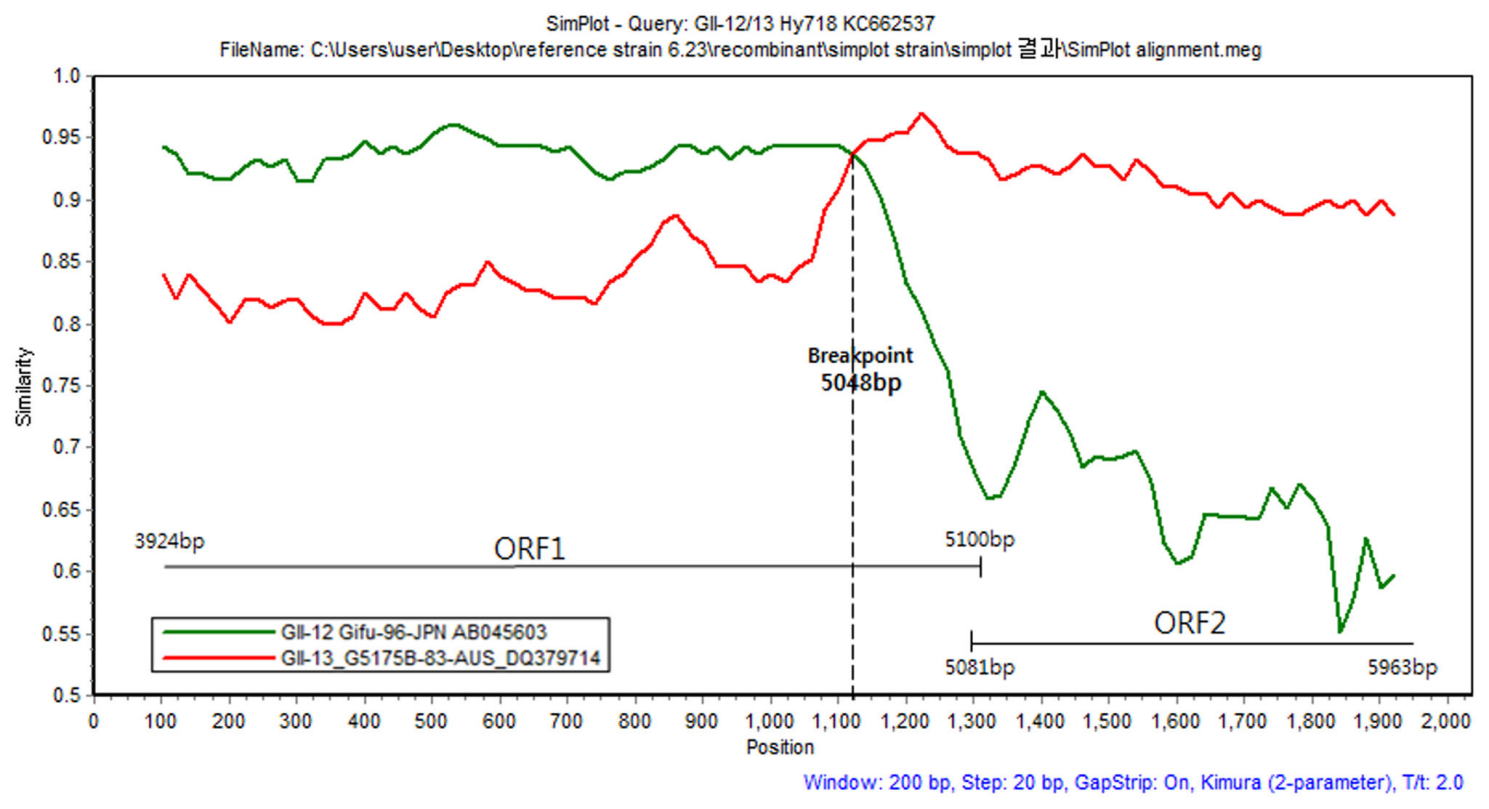
SimPlot analysis was performed using a window size of 200 nucleotides and a 20 nucleotide step. The Hy718-KOR (KC662537) strain was compared with Gifu-96-JPN (green line) and G5175B-83-AUS (red line) using a, partial sequence (2034 bp) of ORF1 and ORF2 regions. The vertical axis indicates the similarity (%) of nucleotide sequence between the query strain and other reference strains. The horizontal axis indicates the positions of nucleotides.

The C9-439 strain (KF289337) was found to share its genome with two other genotypes: the GII-12 capsid region and the GII-13 RdRp region. SimPlot analysis was performed to more accurately investigate the recombination breakpoint. This analysis employed a fragment sequence (3928 - 5963 bp) of part of the capsid and RdRp regions of the GII-12 Gifu-96-JPN strain (AB045603) and the GII-13 G5175B-83-AUS strain (DQ379714). SimPlot data revealed that at position 101 - 1001, the C9-439 strain (KF289337) showed 93.6% similarity to the GII12 Gifu-96-JPN (AB045603) strain and 83.3% similarity to the GII-13 G5175B-83-AUS (DQ379714) strain. The GII-12 Gifu-96-JPN (AB045603) and GII-13 G5175B-83-AUS (DQ379714) strains showed the highest similarity to the C9-439 strain (KF289337) strain at position 1121 (93.78% and 93.76%, respectively). At position 1121 - 1921 of the C9-439 (KF289337) and reference strains, the average similarity was 69.6% for the GII-12 Gifu-96-JPN strain (AB045603) and 91.8% for the GII-13 G5175B-83-AUS strain (DQ379714). These results suggested that recombination occurred at position 1121, which is known to be the ORF1 region of NoV (Figure 6).

## Discussion

NoV are a major cause of gastroenteritis epidemics. Emergence of new strains of the virus has increased research interest to uncover their molecular epidemiological mechanisms [19]. The NoV genogroups GI, GII, and GIV are recognized as the main contributors to common outbreaks, and are associated with acute gastroenteritis in people of all ages worldwide. Each genogroup is further subdivided into GI.1-14 and GII.1-17 genotypes based on the degree of inter-genotype variation and antigenicity [20]. In the case of Korea, NoV related gastroenteritis has been a major public health concern since the virus was first reported in 2005 [21].

In this study, human NoV was detected in 19 out of 500 clinical samples from Korea, and positive samples were classified by genotyping through BLAST search and phylogenetic analyses. As a result, the NoV genotypes GI-6 (*n* = 2), GII-3 (*n* = 4), GII-4 (*n* = 9), GII-6 (*n* = 3), and GII-12/13 (*n* = 1) were isolated. These results generally supported those of many other studies and suggested that GII-4 was the most prevalent genotype identified worldwide [22]. These GII-4 variant strains have also been identified in South Korea, and are rapidly spreading worldwide, including through India and Australia [12,13]. According to a report published by the Seoul Metropolitan Government Institute of Health & Environment (SIHE), the GII-4 (64%) genotype is the most frequent in South Korea, followed by GII-3 (15%) [23]. Furthermore, most NoV outbreaks that occurred in Europe and Asia after 2002 were caused by GII-4 variant strains [24]. With respect to the GI genogroup, two cases of NoV with GI genotypes were identified in this study, supporting the SIHE study that found a high proportion of GI-4 (1.1%), GI-3 (0.9%), GI-2 (0.6%), and GI-6 (0.4%) genotypes among isolates [23]. 

To date, several previous phylogenetic analyses of NoV have been performed using partial sequencing based on partial nucleotide or deducted amino acid sequences of the virus genome [24–26]. However, in some cases, the true genetic relationship among various NoV strains could not be analyzed due to limited sequence variation of partial sequences of the NoV genome [20,27]. To overcome this problem, phylogenetic analyses were performed based on a full sequence analysis of 1 novel strain, C9-439 (KF289337), which was isolated from stool samples. The full sequence was divided into 3 partial sequences: ORF1 (246 bp), ORF2 (483 bp), and ORF3 (212 bp). Within the GII genogroup, the ORF1 region of the C9-439 strain showed 95.5% identity with the GII-12 Gifu-96-JPN strain (AB045603) isolated from the Gifu Prefectural Institute of Japan [28]. The ORF2 and ORF3 regions of the C9-439 strain with the GII-13 genotype also clustered with the GII-13 G5175B-83-Aus strain (DQ379714) [29], which showed 93% and 92.9% similarity, respectively. The GII-12 and GII-13 genotypes of NoV are relatively rare types, and have not been well studied to date. Therefore, the GII-12 Gifu-96-JPN (AB045603) and GII-13 G5175B 83 Aus (DQ379714) strains, which have the longest sequences in the NCBI database, were selected for comparison with the C9-439 strain isolated in this study. 

Recently, high rates of recombination have been frequently reported and can be attributed to viral evolution or mutations, mostly occurring in the region of the junction of ORF1 and ORF2; such recombinant strains have, caused sporadic cases and outbreaks of acute gastroenteritis [30,31]. In this study, a novel strain, C9-439 (KF289337), was isolated within the GII-13 genotype and was confirmed by genotyping using full genomic sequence analysis. Using the SimPlot program, which enabled a more accurate estimation of the breakpoint, we found that recombination of this strain occurred in the ORF1 region. The C9-439 strain showed specificity to the ORF1 region of the GII-12 genotype and to the ORF2 and ORF3 regions of the GII-13 genotype, suggesting that the recombinant strain C9-439 showed a high level of nucleotide conservation with the GII-12 genotype throughout ORF1. SimPlot analysis also revealed that the recombination occurred at approximately 5,048 bp, which corresponded to the RdRp region of ORF1. This result was consistent with that of previous studies, which demonstrated that most recombination events have crossover points within or around the ORF1 and ORF2 regions [8,32]. Han et al. [33] detected a recombinant strain, GII-4/GII-3, from children with acute gastroenteritis in Korea, which was predominant among the isolated strains. These strains were not found until 2009, and another new recombinant strain, GII-6/GII-14, was detected with a GII-b polymerase and a capsid of GII-1, GII-2, GII-3, or GII-4, indicating changing patterns in the predominant strains of NoV in Korea [33]. In India, intragenotype recombination events were reported in 2010. Although most recombination events to date have occurred either between the ORF1 and ORF2 regions or between the RdRp and ORF2 regions [8], the recombination events of the new recombinant strain (P 51) found in India occurred between the ORF2 and ORF3 regions [25]. In addition, Nataraju et al. [12] detected the Hu/NoV/IDH1501/2009/IND recombinant strains in India using similarity plot and phylogenetic analyses. They also found a novel recombinant strain, Hu/NoV/IDH1873/2009/IND, with a GII-5 like RdRp gene and a GII-13 like capsid gene [12]. As a novel strain, the GII-12/13 strain found in the present study has not yet been studied in detail; however, it should be a focus of further research as it may provide important insights into the mechanisms of NoV recombination. In particular, recombination and mutation in the NoV genome could be important factors in the generation of its genetic diversity [34]. 

 Full sequence analysis is not yet a common technique in molecular epidemiology; therefore, it is not easy to detect these new viruses, and it is also difficult to treat patients whose immune systems have been compromised by infection with new recombinant strains of NoV. Furthermore, recombination of NoV strains could lead to confusion of the whole classification system of the virus. Therefore, genomic studies aimed at investigating the diversity of NoV will play an essential role in elucidating the phylogenetic and evolutionary relationships of the viruses [35]. Partial genomic analysis of NoV may not be sufficient for investigating uncommon and novel strains. On the contrary, our full genome sequence information is enables identification of the origin of a strain and its genetic relationship to other circulating strains [36]. Full sequence analyses of human NoV are relatively rare in Korea; however, NoV full genome sequencing has been conducted in approximately 100 strains isolated worldwide [35]. 

The wide genetic diversity of human NoV has been reported in different geographic locations and at various times [17]. These variants could be related to antigenic variations that alter viral transmission and immune systems in human bodies, thus influencing the patterns of viral activities [37,38]. Therefore, studies of the genetic diversity and evolution of human NoV could provide important information that may prove useful for controlling human NoV infection [26]. Here, we determined the full-length sequences of a recombinant NoV strain isolated from clinical samples in South Korea. Because this novel recombinant strain may result in hazardous NoV outbreaks in Korea, this information should prove to be valuable. 

In conclusion, the present study isolated NoV from acute gastroenteritis patients and identified a novel recombination strain, which was derived from a rare type of NoV. The results from this study highlight the many challenges in the identification of new recombination strains and suggest that guidelines be applied for identifying newly emerging recombinant strains of NoV. 
